# Barriers and facilitators to the integration of digital technologies in mental health systems: A protocol for a qualitative systematic review

**DOI:** 10.1371/journal.pone.0259995

**Published:** 2021-11-22

**Authors:** Chiara Berardi, Madeleine Hinwood, Angela Smith, Adrian Melia, Francesco Paolucci

**Affiliations:** 1 Newcastle Business School, The University of Newcastle, Newcastle, Australia; 2 School of Medicine and Public Health, The University of Newcastle, Callaghan, New South Wales, Australia; 3 Hunter Medical Research Institute, New Lambton Heights, NSW, Australia; 4 Hunter New England Health Libraries, Hunter New England Local Health District, New Lambton, New South Wales, Australia; Stellenbosch University and Kenya Medical Research Institute, KENYA

## Abstract

**Introduction:**

Digital technology has the potential to improve health outcomes and health system performance in fragmented and under-funded mental health systems. Despite this potential, the integration of digital technology tools into mental health systems has been relatively poor. This is a protocol for a synthesis of qualitative evidence that will aim to determine the barriers and facilitators to integrating digital technologies in mental health systems and classify them in contextual domains at individual, organisational and system levels.

**Methods and analysis:**

The methodological framework for systematic review of qualitative evidence described in Lockwood et al. will be applied to this review. A draft search strategy was developed in collaboration with an experienced senior health research librarian. A systematic search of Medline, Embase, Scopus, PsycInfo, Web of Science and Google Scholar, as well as hand searching of reference lists and reviews will identify relevant studies for inclusion. Study selection will be carried out independently by two authors, with discrepancies resolved by consensus. The quality of selected studies will be assessed using JBI Critical Appraisal Checklist for Qualitative Research. Data will be charted using JBI QUARI Data Extraction Tool for Qualitative Research. Findings will be defined and classified both deductively in a priori conceptual framework and inductively by a thematic analysis. Results will be reported based on the Enhancing transparency in reporting the synthesis of qualitative research. The level of confidence of the findings will be assessed using GRADE-CERQual.

**Ethics and dissemination:**

This study does not require ethics approval. The systematic review will inform policy and practices around improving the integration of digital technologies into mental health care systems.

## Introduction

### Background

Mental health disorders are associated with significant health, social, and economic consequences, including increased morbidity and mortality rates, social exclusion, low productivity, unemployment and lower education levels [1]. In spite of the significant direct and indirect impacts of mental health disorders for individuals and communities, there is an imbalance between the burden of mental health disorders and allocation of resources [2–4]. This results in generally worse access and availability of mental health care, compared with care for physical conditions [2, 5]. Internationally, mental health care tends to be fragmented, under-funded, and managed quite separately from physical health [2].

The expansion of digital technologies in health care generally, such as electronic health records, telemedicine, and applications on smartphones [6], has also provided innovative methods for supporting and delivering mental health care. Multiple recent reviews for delivering mental health care via digital technologies have shown that these are safe, feasible and effective among groups of people with mental health disorders [7–13]. Furthermore, digital technologies have the potential to improve mental health care systems, increase system capacity and transparency [14], enable data collection for monitoring and measure health outcomes, facilitate data sharing among health services [15, 16], enhance access in rural and remote areas [17–20], and improve rationing of costs and human resources [14, 16]. The integration and reimbursement of digital technology tools at different levels of the health systems depends on the performance and capacity to meet health system objectives such as quality, accessibility, efficiency and equity [21]. This potential for the strengthening of the health system is recognised in the WHO global strategy of digital health 2020–2025, which provides recommendations to integrate digital health strategies across health systems [6]. Collectively, these developments highlight the promise of digital technologies to improve quality of and access to mental healthcare. Indeed, both patients’ health outcomes and mental healthcare system performance can benefit from the digitalisation of mental health care.

### Rationale

Despite the potential of digital technologies for shaping and improving mental healthcare systems, to date the integration of these services has been limited [22–26]. This is likely to be due to a range of factors, including the lack of regulatory frameworks [27], limitations in health system infrastructure and governance [28], ethical and privacy standards [29], limited understanding of the potential impact upon the quality of clinical care [30], and individual preferences [31, 32]. The COVID-19 pandemic was a recent exception, as the acceptance and integration of technologies for the delivery of mental healthcare rapidly accelerated in order to deliver mental healthcare services remotely and maintain social distancing recommendations [33, 34]. Although massively expanded access to digital technologies observed during the height of the pandemic, which tended to be funded by short-term emergency financing mechanisms, is unlikely to continue to the same extent post-pandemic. The increased use of digital technologies across service types in response to the pandemic provided an example of how digital technologies can be integrated to meet the needs of health systems and patients.

Recognising the barriers and facilitators to the integration of digital technologies processes and interventions is therefore important to improve access to these technologies, which have repeatedly been shown to hold enormous benefit for patients, providers, and health systems. The need for this is evident in national and international policy [6, 21], health care system reviews [35], clinical guidelines [36] and allocation of research funds [37], which collectively call for greater investment and capacity building in digital technologies. Identifying barriers and facilitators to integrating digital technologies in health systems is therefore important to inform these initiatives [38–40].

The qualitative systematic review described in this protocol therefore aims to determine the barriers and facilitators to the integration of digital technologies, taking into account the complexity of mental healthcare systems [41]. We will therefore identify barriers and facilitators across levels of the mental healthcare systems both inductively, using a thematic analysis and deductively, using the Furst et al. [42] ecosystem approach to mental health research to define different domains. A modified version of Tansella and Thornicroft’s matrix [43] proposed by Furst et al. [44] will be used to define the mental healthcare system levels. A comprehensive understanding of the nature of these barriers and facilitators, mapped to these frameworks [42, 43] will further the development of targeted solutions, informing policy design and innovation to improve mental healthcare systems at all levels.

To our knowledge this will be the first qualitative systematic review on the barriers and facilitators to the integration of digital technologies in mental health systems. Previous reviews in this field have focused on: (i) the effectiveness of digital technologies for the treatment of mental health disorders [7–11]; (ii) health or mental health systems’ needs, challenges and barriers, without including digital technologies [45, 46]; (iii) financial and health resource benefits that digital technologies provide to healthcare settings [14, 47]; (iv) barriers and facilitators to the integration of e-services, without a specific focus on mental health settings [48], and (v) the optimisation of technology integration in primary mental health care [49].

## Methods and analysis

A qualitative systematic review method was chosen to navigate contextual barriers and facilitators to integration of digital technologies in mental health system and to inform a range of specific questions by a systematic search. To develop our qualitative systematic review methodology, we used the Joanna Briggs Institute (JBI) framework of systematic reviews of qualitative evidence by Lockwood et al. [50], and Preferred reporting items for systematic review and meta-analysis protocols (PRISMA-P) 2015 statement [51] (see S1 Table in S1 Appendix). Accordingly, the protocol defines the following steps:

1) Research questions and objectives of the review; 2) Eligibility criteria; 3) Search strategy and study selection; 4) Assessment of methodological quality; 5) Data extraction; 6) Data synthesis. Additionally, this protocol presents a priori categorisation framework to classify the findings of the qualitative systematic review, provides a discussion section and reports administrative supporting information.

The preliminary search was run on 19^th^ of February 2021 and the review is expected to be completed in February 2022.

### Step 1: Research questions and objectives of the review

We developed the research questions following the Population, Phenomena of Interest, Context (PICo) mnemonic, according to the JBI framework for qualitative systematic reviews. The aim of the qualitative systematic review is to answer the following research questions:

1. What are the contextual barriers and facilitators to integration of digital technologies across different levels of mental healthcare systems?
2. How can these findings inform digital technologies research and policymaking in mental health systems?

With this protocol, we define the rationale and a priori methodology for the qualitative systematic review [51], with the following objectives:

- Determine the barriers and facilitators to integrating digital technologies for the treatment and management of mental health care into mental health systems.
- Compare barriers and facilitators to the integration of digital technologies in mental health systems in countries with different contextual factors;
- Identify the barriers and facilitators across different domains and levels of mental health systems;
- Assess the quality of existing literature evidence and developing consistent terminology and systematic approaches to inform future research;
- Make targeted recommendations for policy, implementation, and future research around supporting the integration of digital technologies in mental health systems, based on the review findings.

### Step 2: Eligibility criteria

#### Types of studies

Studies will be considered if they are primary research articles which include qualitative data around barriers and facilitators of the integration of digital technology tools in mental health systems including, but not limited to, designs such as ethnography, action research, case studies, implementation studies, qualitative process evaluation, qualitative interviews with stakeholders. Moreover, mixed method studies will be included if they provide relevant qualitative components and findings reported separately from quantitative findings.

#### Population

We will include studies examining digital technologies as defined by the WHO Global strategy on digital health 2020–2025 [6] which includes: internet of things, virtual care/telemedicine, remote monitoring, artificial intelligence, smart wearables, platforms, big data analytics and tools enabling data storage, remote capture, sharing and exchange across the mental health system. We adopt the WHO definition as it sets strategic policy actions at global level for the next five years. Moreover, it offers a standardised and comprehensive list of terms, which may assist in overcoming the heterogeneity of terminology in the emerging digital health landscape.

#### Phenomena of interest

We will select studies that outline barriers, and/or facilitators to the integration of digital technology categorised under different domains as informed by Furst et al. [42, 44] eco-system approach to mental health research. If some barriers identified during the study selection cannot be categorised under this list of domains (a) places and communities’ features, such as natural, social and human capital that include infrastructure, institution and governance (b) social, demographic, and environmental determinants of health, (c) behaviours and lifestyles and (d) integrated care provision [41], additional categories will be integrated by a thematic analysis to complement the one listed above.

#### Context

The barriers will be identified across different levels of the mental health systems as defined and drawn from Furst et al. [42, 44]. High-, middle- and low-income countries literature will be included in the qualitative systematic review.

### Step 3: Search strategy and study selection

We will develop a search strategy in consultation with a medical librarian to identify a comprehensive search of published literature around multi-domain and multi-level barriers and facilitators to the integration of digital technology in mental health systems. We will refer to other systematic reviews in similar areas [45, 46, 48]. The initial search strategy was developed in Medline using three broad concepts: digital health, barriers and facilitators, and mental health systems. The initial search was conducted on the 19^th^ of February 2021 in Medline, and 2472 references were identified and imported in Endnote (see S2 Table in S1 Appendix). After excluding duplications, 2347 references were included. The Medline search will be extended and adapted to Embase, Scopus, PsycInfo, and Web of Science. The searches in these databases will be complemented by a screening of the first 200 Google Scholar citations, based on the Medline strategy. The searches will be limited to English language peer-reviewed references published between January 2010 and March 2021. The field of digital health technologies is rapidly evolving, and this review will aim to capture current information. In 2010 the rate of disruptive technologies started to grow rapidly and their integration in the healthcare sectors become inevitable [52]. Therefore, the review will be limited to contemporary studies published within the last decade (2010 to 2021), a time frame that has been applied previously for systematic reviews on digital health technologies for mental illness [53, 54]. Table 1 outlines type of studies dates, publication type, language limitation and the proposed inclusion and exclusion criteria, following the JBI PICo categories for systematic reviews of qualitative evidence [50, 55].

**Table 1:** Inclusion and exclusion criteria.

	Inclusion	Exclusion
**Population**	Internet of things, virtual care, remote monitoring, artificial intelligence, smart wearables, platforms, big data analytics and tools enabling data storage, remote capture, sharing and exchange	Any other intervention
**Phenomena of Interest**	Barriers to different domains: (a) places and communities’ features, such as infrastructure, institutions, and governance; (b) social, demographic, and environmental determinants of health; (c) behaviours and lifestyles and (d) integrated care provision and facilitators.	Nil exclusion criteria
**Context**	All stakeholders in different levels of mental health systems and services: (1) nano (patient-professional), (2) micro (facilities, services, teams), (3) meso (patient-organisation) and (4) macro (regional or country level relationship among organisations). High-, middle- and low-income countries.	Any other health setting
**Types of studies**	Primary research of qualitative findings: ethnography, action research, case studies, implementation studies, qualitative process evaluation, qualitative interviews with stakeholders.	Quantitative studies, Comment or editorial or letter or news
**Timing**	1^st^ Jan 2010- 4^th^ March 2021	Before 1^st^ Jan 2010 and after 4^th^ March 2021
**Language**	English	Any other language
**Publication type**	Peer reviewed	No peer reviewed

Studies identified in the search will be collated in EndNote X9 and exported to Covidence data management software for screening of titles and abstracts, and full-text reviews. All titles and abstracts will be independently reviewed by two authors according to the proposed eligibility criteria in Table 1. Any conflicts will be resolved in consultation with a third author. Where consensus is not reached, the article will be automatically progressed to the next stage of the review. Following title and abstract screening, full texts of potentially relevant studies will be assessed for eligibility by two authors independently, with any conflicts resolved by consensus among authors. Review authors will communicate regularly during the screening process to discuss study selection, with any alterations recorded. The selection process will be reported using a PRISMA flow diagram [50, 55].

### Step 4: Assessment of methodological quality

As we aim to select primary research studies using qualitative data, critical quality appraisal of the final articles selected will be performed using JBI Critical Appraisal Checklist for Qualitative Research [56] (see S3 Table in S1 Appendix). Two authors will critically appraise all included papers in order to determine the overall quality of available evidence. As this review aims to inform policy making, studies that rate critically low quality will be excluded and discussed in a separate paragraph.

### Step 5: Data extraction

An initial pilot trial of data extraction will be performed in consultation with all authors, using a sample of the final included studies of a variety of qualitative designs, to assure comprehensive and satisfactory inclusion of relevant, consistent and reliable data. During the piloting process, any changes or additional items that are relevant with the aims of this study will be added to the initial data charting format and applied to all the selected studies. We will develop a standardised data extraction form in Excel to chart the data based on the JBI QUARI Data Extraction Tool for Qualitative Research [50] (see S3 Table in S1 Appendix). A level of credibility will be assigned to each reference as provided by the JBI guidelines for qualitative review [50] (see S4 Table in S1 Appendix). We will integrate this tool with additional information guided by the a priori categorisation framework proposed by Furst et al. [42, 44]. A draft version of the items, that will be included in the data extraction form, is presented in Table 2. For each source, data will be charted by one author and checked by a second author. Authors will read full text reference and highlight the extracted data. Each finding will be reported with illustration from the publication [50]. Any discrepancies will be resolved by consensus among authors. Reporting data will include also gaps, limitations and quality assessment of the selected literature on the topic.

**Table 2:** Initial data extraction form.

Category	Variable
**General information on the reviewer**	Reviewer
	Date
**General information of the publication**	Bibliographic information (Author, Year, Journal Record number)
	Country of publication and conduct
**Study Description**	Aim
	Methodology
	Methods
	Setting
	Geographical
	Cultural
	Participants (general population, demographic, epidemiological features etc.)
	Data analysis
	Authors conclusions
	Comments
	Complete (Yes/No)
**Population**	Definition of digital technology
	Technology features and options
**Phenomena of Interest**	Definition of barriers and facilitators
	Domain’s categories and subcategories of barriers and facilitators
**Context**	Mental health system levels
	Stakeholders involved (patients, provides, organisations, administrators policy makers, governments)
**Findings**	Illustration from Publication (page number)
	Level of credibility (QUADRI Data Extraction Tool for Qualitative Research (Unequivocal, Credible, Unsupported) Extraction findings complete (Yes/No)
**Gaps and limitations**	Standardisation and terminology accuracy
	Qualitative assessment of studies
	Reported Limitations—studies
	Other identified limitations
**Quality assessment (JBI critical appraisal tools)**	High
	Medium
	Low
	Critically low
**Additional information**	Any information relevant to the research question

### Step 6. Data synthesis

We will report results based on the Enhancing transparency in reporting the synthesis of qualitative research (ENTREQ) [57]. The goal of data synthesis will be to report and illustrate the barriers and facilitators to the integration of digital technologies in mental healthcare as closely as possible to their original description. To develop some organisation of the findings the domains of barriers and facilitators will be defined both deductively in an a priori conceptual framework proposed by Furst et al. [42, 44] and inductively by a thematic analysis [58]. Domains of barriers and facilitators defined a priori framework will be integrated by analytical domains defined by the thematic analysis to ensure consistency among reported findings, avoid misinterpretation of relevant data and assure that all relevant domains are captured [58]. Thematic analysis will be performed EPPI-Reviewer using methods described by Thomas et al. [58] which provides three steps: 1) definition of free line-by-line coding of primary studies’ findings; 2) organisation of free codes to define descriptive themes; 3) construction of analytical themes. For each domain of barriers and solutions, similar findings will be aggregated and accompanied by an inclusive statement representing all the findings of the specific domain. Each domain will include at least 2 findings based on similarity of concept. If pooling in aggregated domains for some of the items charted is not possible, findings will be descriptively narrated [50]. To provide robust policy recommendations, an indication on the level of confidence of our findings will be reported using GRADE-CERQual [59, 60], focusing on methodological limitations [61], coherence [62], adequacy of data [63], relevance of data [64] and dissemination bias [65]. A level of certainty (“high”, “moderate”, “low” or “very low”) will be assigned to each component of the findings [59]. The domains and the level of credibility of findings will be defined and reported by one author and checked by a second author with discrepancies resolved by consensus.

#### A priori categorisation framework

We will use the healthcare ecosystem approach to mental health research developed by Furst et al. [42] as a priori framework to categorise the multi-disciplinary nature of barriers and facilitators across different domains and levels of the health care system. This includes four main domains: the places and communities in which we live, including infrastructure, institutions and governance; the broader determinants of health (such as the social and demographic characteristics of the environment); health behaviours and lifestyles; and integrated healthcare provision [41, 42, 44], mapped across different levels of the healthcare system (nano (patient–professional level), micro (service level), meso (local area/organisation level) and macro (region/country level)). Acknowledging the contextual factors that may affect the digitalisation process of mental health care across the domains and levels of a population health system are likely to be particularly relevant for the translation of knowledge into policy and practice [4, 42, 44].

## Discussion

The qualitative systematic review will summarise current literature concerning the domains of barriers to the integration of digital technology and potential facilitators in mental health settings. Digital technologies offer sustainable solutions for health systems to improve coverage and reduce fragmentation, however they have generally experienced difficulties in achieving integration. Our findings will provide a comprehensive summary of the barriers and facilitators to integrating digital technologies into the mental health systems of high and low-middle income countries, using a framework designed to delineate domains and levels of the mental healthcare system [42, 44]. Due to the complexity and implications of the digitalisation process in mental healthcare systems, the qualitative systematic review will aim to identify the interactions in terms of barriers and facilitators across different functions, organisations and actors involved to inform future policy and practice.

### Strengths and limitations

To our knowledge, this study is the first review on barriers and facilitators to digital technologies in mental health systems. We acknowledge that relevant non-English and emerging grey literature in this area might be missing. As we are not including grey literature, potentially relevant publications, including reports by organisations and governments, may be missed. Any additional limitations identified throughout the qualitative systematic review will be acknowledged in the publication of the review.

## Supporting information

S1 Appendix (DOCX)
