# Supplementary material for: Barriers and facilitators to the integration of digital technologies in mental health systems: A protocol for a qualitative systematic review
Source: PLoS One. 2021 Nov 22;16(11):e0259995. doi: 10.1371/journal.pone.0259995 (PMC8608309; doi:10.1371/journal.pone.0259995)

**S1 Appendix**

**S1 Table:** Preferred reporting items for systematic review and meta-analysis protocols (PRISMA-P) 2015 statement. Systematic Reviews 2015 4:1

| **Section/topic** | **#** | **Checklist item** | **Information reported** | | **Line number(s)** |
| --- | --- | --- | --- | --- | --- |
|  |  |  | **Yes** | **No** |  |
| **ADMINISTRATIVE INFORMATION** | | | | | |
| **Title** | | | | | |
| Identification | 1a | Identify the report as a protocol of a systematic review |  |  | Line 1-3, Page 1 |
| Update | 1b | If the protocol is for an update of a previous systematic review, identify as such |  |  | N/A |
| **Registration** | 2 | If registered, provide the name of the registry (e.g., PROSPERO) and registration number in the Abstract |  |  | Line 53, Page 2 |
| **Authors** | | | | | |
| Contact | 3a | Provide name, institutional affiliation, and e-mail address of all protocol authors; provide physical mailing address of corresponding author |  |  | Line 5-22,  Page 1 |
| Contributions | 3b | Describe contributions of protocol authors and identify the guarantor of the review |  |  | Line 326-329,  Page 15 |
| **Amendments** | 4 | If the protocol represents an amendment of a previously completed or published protocol, identify as such and list changes; otherwise, state plan for documenting important protocol amendments |  |  | N/A |
| **Support** | | | | | |
| Sources | 5a | Indicate sources of financial or other support for the review |  |  | Line 330-332,  Page 15 |
| Sponsor | 5b | Provide name for the review funder and/or sponsor |  |  | Line 330-332,  Page 15 |
| Role of sponsor/funder | 5c | Describe roles of funder(s), sponsor(s), and/or institution(s), if any, in developing the protocol |  |  | Line 330-332,  Page 15 |
| **INTRODUCTION** | | | | | |
| **Rationale** | 6 | Describe the rationale for the review in the context of what is already known |  |  | Line 87-129,  Page 4-5 |
| **Objectives** | 7 | Provide an explicit statement of the question(s) the review will address with reference to participants, interventions, comparators, and outcomes (PICO) |  |  | Line 147-167  Page 6-7 |
| **METHODS** | | | | | |
| **Eligibility criteria** | 8 | Specify the study characteristics (e.g., PICO, study design, setting, time frame) and report characteristics (e.g., years considered, language, publication status) to be used as criteria for eligibility for the review |  |  | Line 169-200,  Page 7-8 |
| **Information sources** | 9 | Describe all intended information sources (e.g., electronic databases, contact with study authors, trial registers, or other grey literature sources) with planned dates of coverage |  |  | Line 205-219,  Page 9 |
| **Search strategy** | 10 | Present draft of search strategy to be used for at least one electronic database, including planned limits, such that it could be repeated |  |  | Line 202-209,  Page 9,  Table S2 Appendix |
| ***STUDY RECORDS*** | | | | | |
| Data management | 11a | Describe the mechanism(s) that will be used to manage records and data throughout the review |  |  | Line 224-225, 232-233, Page 10-11 |
| Selection process | 11b | State the process that will be used for selecting studies (e.g., two independent reviewers) through each phase of the review (i.e., screening, eligibility, and inclusion in meta-analysis) |  |  | Line 225-232, Page 10-11 |
| Data collection process | 11c | Describe planned method of extracting data from reports (e.g., piloting forms, done independently, in duplicate), any processes for obtaining and confirming data from investigators |  |  | Line 243-247, Page 11 |
| **Data items** | 12 | List and define all variables for which data will be sought (e.g., PICO items, funding sources), any pre-planned data assumptions and simplifications |  |  | Line 247-254, Table 2, Page 12 |
| **Outcomes and prioritization** | 13 | List and define all outcomes for which data will be sought, including prioritization of main and additional outcomes, with rationale |  |  | N/A |
| **Risk of bias in individual studies** | 14 | Describe anticipated methods for assessing risk of bias of individual studies, including whether this will be done at the outcome or study level, or both; state how this information will be used in data synthesis |  |  | Line 234-240; Page 11 |
| ***DATA*** | | | | | |
| **Synthesis** | 15a | Describe criteria under which study data will be quantitatively synthesized |  |  | N/A |
|  | 15b | If data are appropriate for quantitative synthesis, describe planned summary measures, methods of handling data, and methods of combining data from studies, including any planned exploration of consistency (e.g., *I* ^2^, Kendall’s tau) |  |  | N/A |
|  | 15c | Describe any proposed additional analyses (e.g., sensitivity or subgroup analyses, meta-regression) |  |  | N/A |
|  | 15d | If quantitative synthesis is not appropriate, describe the type of summary planned |  |  | Line 265-280, Page 13 |
| **Meta-bias(es)** | 16 | Specify any planned assessment of meta-bias(es) (e.g., publication bias across studies, selective reporting within studies) |  |  | N/A |
| **Confidence in cumulative evidence** | 17 | Describe how the strength of the body of evidence will be assessed (e.g., GRADE) |  |  | Line 280-286, Page 13 |

**S2 Table:** Medline search strategy

**Medline**

Database: Ovid MEDLINE(R) and Epub Ahead of Print, In-Process & Other Non-Indexed Citations and Daily <1946 to February 19, 2021>

Search Strategy:

--------------------------------------------------------------------------------

1 telemedicine/ or telerehabilitation/ (26923)

2 (telemedicine or tele-medicine or telemental or tele-mental or telehealth or tele-health or telepsychiatr* or tele-psychiatr*).ti,ab,kw,kf. (21389)

3 biomedical technology/ or health technolog*.ti,ab,kw,kf. (14511)

4 (digital adj2 (health or technolog* or revolution or intervention*)).ti,ab,kw,kf. (7140)

5 emerging technolog*.ti,ab,kw,kf. (6517)

6 (ehealth or e-health or e-mental or e-therap* electronic health or mhealth or m-health or mobile health).ti,ab,kw,kf. (15545)

7 mobile applications/ (7005)

8 (app or apps or app-based or appbased).ti,ab,kw,kf. (31787)

9 (mobile adj2 application*).ti,ab,kw,kf. (4865)

10 internet/ or internet-based application/ (74975)

11 (website* or web-site* or web-based or internet or internet-based or online or on-line).ti,ab,kw,kf. (253065)

12 1 or 2 or 3 or 4 or 5 or 6 or 7 or 8 or 9 or 10 or 11 (368193)

13 mental health/ (41778)

14 mental disorders/ (165580)

15 (mental health or mental healthcare or behavio?ral health or mental disorder* or mental illness*).ti,ab,kw,kf. (228906)

16 mental health services/ or community mental health services/ (52974)

17 13 or 14 or 15 or 16 (362997)

18 "delivery of health care"/ or health care reform/ or health services accessibility/ or (reform or reforms or reforming).ti,ab,kw,kf. (220527)

19 Health Policy/ or (policy or policies).ti,ab,kw,kf. (314190)

20 health services research/ (37357)

21 global health/ or (global or globally).ti,ab,kw,kf. (499349)

22 economics/ or "cost and cost analysis"/ or health care costs/ or health expenditures/ or (cost* or expenditure* or financ* or reimbursement* or economic* or austerit* or fund or funded or funding or underfund* or fragment* or welfare).ti,ab,kw,kf. (1523184)

23 (deliver* adj3 (care or healthcare or system*)).ti,ab,kw,kf. (133709)

24 (healthcare system* or health care system* or health system* or mental healthcare system* or "mental health care system*").ti,ab,kw,kf. (123210)

25 "health service needs and demand"/ or needs assessment/ or (assess* adj1 need*).ti,ab,kw,kf. (37279)

26 18 or 19 or 20 or 21 or 22 or 23 or 24 (2471267)

27 25 and 26 (12824)

28 26 or 27 (2471267)

29 12 and 17 and 28 (5016)

30 ((implement* or innovat* or integrat* or uptake* or up-tak* or accept* or scal* up or scal*-up or up-scal* or adher* or promot* or adopt* or engag* or improvement* or intervention*) and (barrier* or impede or impediment* or facilitat* or challeng* or solution* or driver* or optimi* or diffus* or infus* or success* or limitation* or strateg* or approach* or hinder* or obstacle* or hurdle* or opportunit* or enabl* or sustain* or operational* or adapt* or capacit* or polic*)).ti,ab,kw,kf. (2222549)

31 29 and 30 (2701)

32 limit 31 to yr="2010 -Current" (2553)

33 limit 32 to (comment or editorial or letter or news) (30)

34 32 not 33 (2523)

35 limit 34 to english language (2472)

**S3 Table**: JBI Appraisal Checklist for Qualitative Research


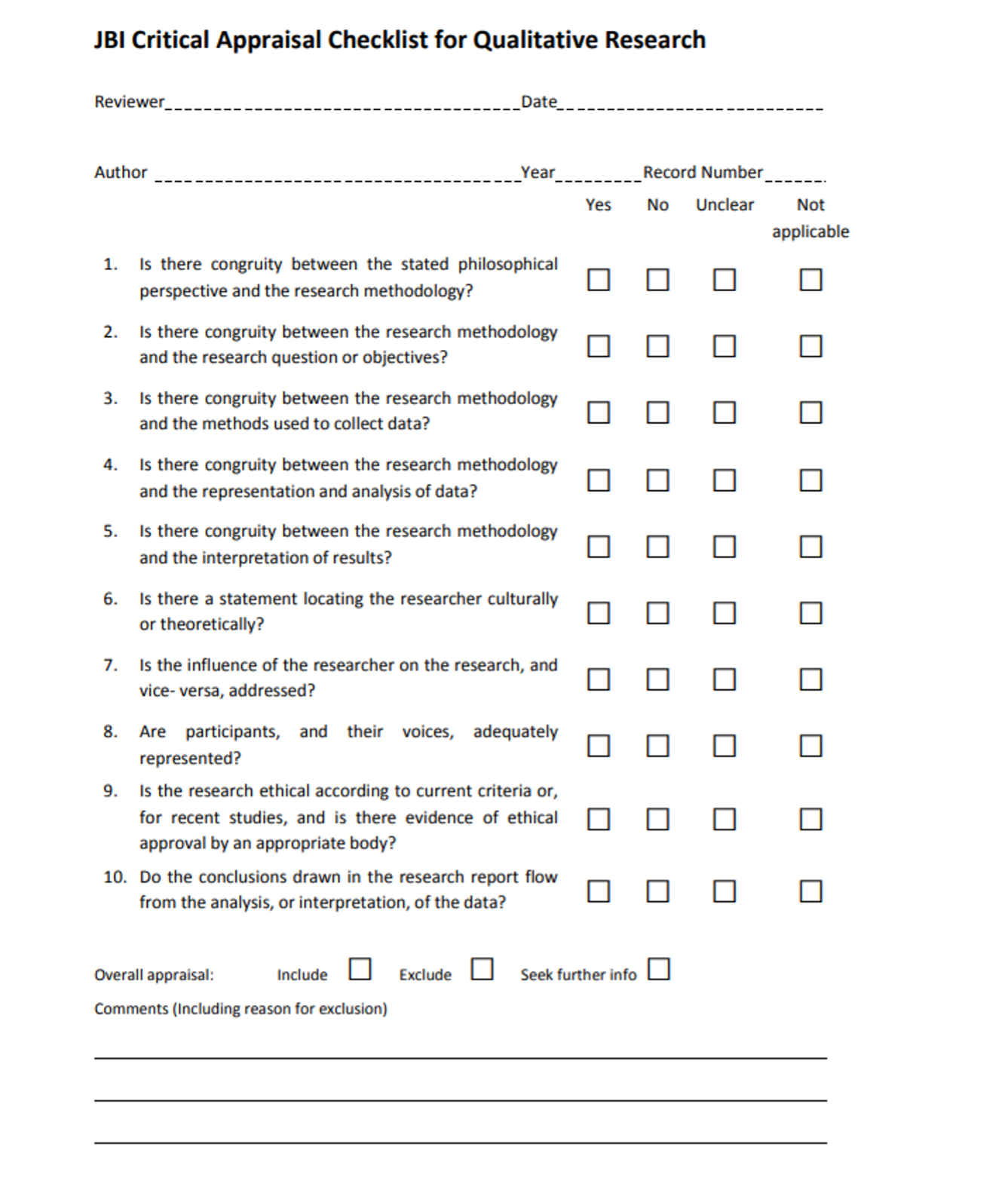


**S4 Table:** JBI QUARI Data Extraction Tool for Qualitative Research


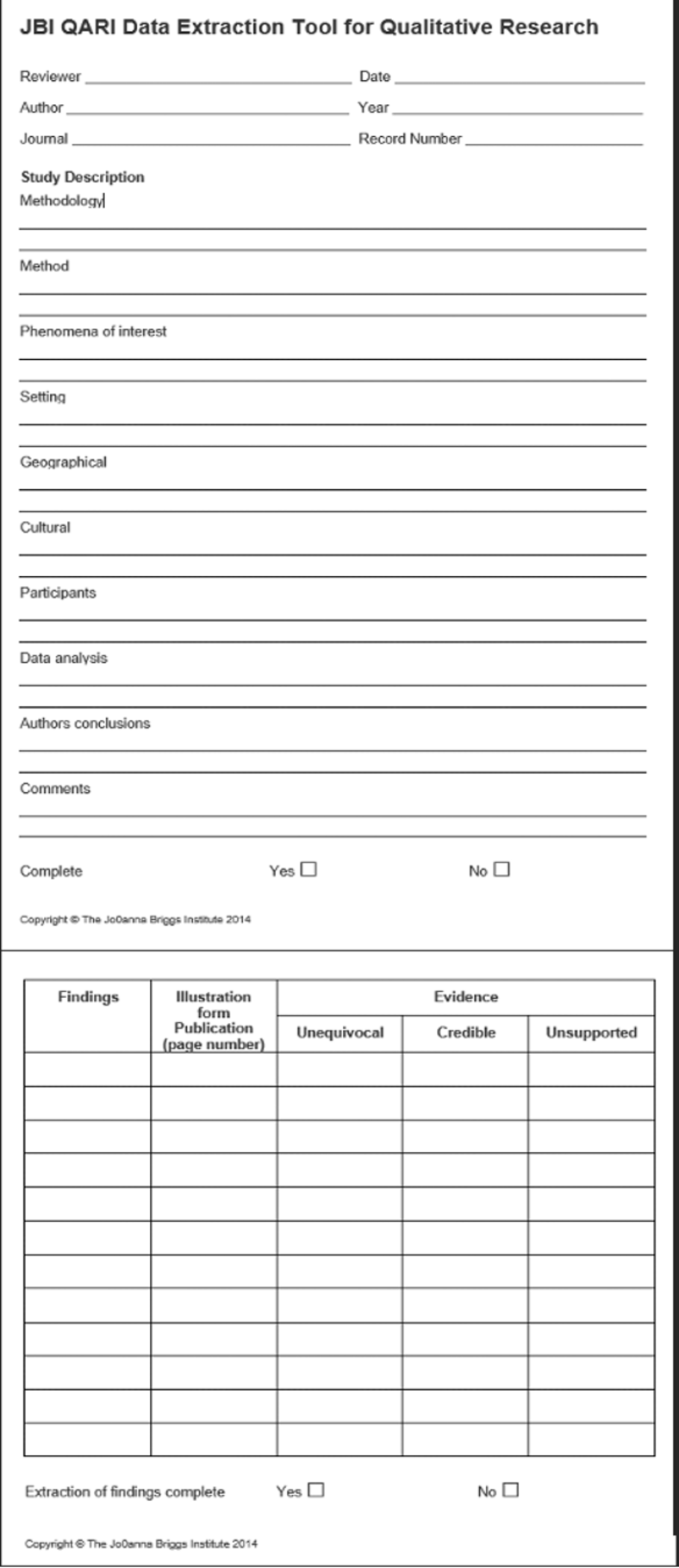

Supplement: S1 Appendix — (DOCX) [file pone.0259995.s001.docx]
